# SeuratIntegrate: an R package to facilitate the use of integration methods with Seurat

**DOI:** 10.1093/bioinformatics/btaf358

**Published:** 2025-06-23

**Authors:** Florian Specque, Aurélien Barré, Macha Nikolski, Domitille Chalopin

**Affiliations:** CNRS UMR 5095, IBGC, Biological and Medical Sciences Department, University of Bordeaux, Bordeaux F-33000, France; Bordeaux Bioinformatics Center (CBiB), University of Bordeaux, Bordeaux 33076, France; CNRS UMR 5095, IBGC, Biological and Medical Sciences Department, University of Bordeaux, Bordeaux F-33000, France; Bordeaux Bioinformatics Center (CBiB), University of Bordeaux, Bordeaux 33076, France; CNRS UMR 5095, IBGC, Biological and Medical Sciences Department, University of Bordeaux, Bordeaux F-33000, France

## Abstract

**Motivation:**

Integrating multiple datasets has become an increasingly common task in scRNA-seq analysis. The advent of single-cell atlases adds further complexity, as they often involve combining data with nested batch effects. While common tools such as Seurat offer access to batch-correction methods, the diversity of available options remains limited. With growing evidence that integration method performance varies significantly between datasets, making an informed decision in selecting the most appropriate integration approach is not trivial. A broader range of accessible methods combined with a comprehensive toolbox for comparative integration analysis, would support more effective and flexible single-cell data integration workflows.

**Results:**

Built on Seurat’s foundations, we developed SeuratIntegrate, an open source R package that expands integration methods available to Seurat users, including Python-based approaches, while operating entirely within the R environment. The package enables integration benchmarking using well-established performance metrics, and provides automated Python environment management, cross-language object conversion, and tools for score handling and visualization. All features are designed for ease of use and extensibility.

**Availability and implementation:**

The source code, installation process and vignettes demonstrating usage are freely available on GitHub: https://github.com/cbib/Seurat-Integrate. A Zenodo deposit contains a copy of the package code along with the data to reproduce the results presented above (accession 10.5281/zenodo.14288360). The package is released under the MIT License.

## 1 Introduction

The rapid growth of single-cell datasets in recent years has enabled the possibility to study multiple samples from different biological and technical contexts, all aggregated in large atlases. These atlases compile data from different experiments, reagent batches or sequencing technologies. While these resources are invaluable for advancing single-cell based studies, bringing together such large datasets presents the challenge of overcoming confounding effects that may mask true biological differences and thus potentially impact analyses such as for example differential expression (Argelaguet *et al.* 2021, Nguyen *et al.* 2023, Maan *et al.* 2024).

Seurat v5 (Hao *et al.* 2024) and Scanpy (Wolf *et al.* 2018) are the most widely used R-based and Python-based tools, respectively, for single-cell data analysis. Both tools offer comprehensive functionalities, including visualization, clustering, and trajectory analysis. A key feature of both is their support for batch-effect correction and data integration, essential steps for harmonizing datasets from diverse biological and technical contexts. Particularly, Seurat v5 streamlines integrative analyses by introducing a multi-layered object structure enabling batch-wise normalization and a unified and consistent interface to multiple integration algorithms.

Seurat originally adopted an “anchor-based” strategy for integration based on Mutual Nearest Neighbors (MNN, Haghverdi *et al.* 2018) for batch-effect correction. Version 5 added native support for Harmony (Korsunsky *et al.* 2019) and the Python-based scVI (Lopez *et al.* 2018), bringing the total number of supported integration methods to five. However, Luecken *et al.* (2022) evaluated the performance, usability, and scalability of over 20 integration and batch-effect correction methods and demonstrated that their effectiveness varies widely across contexts depending on data complexity, such as the number of samples, cell-type diversity, and technological differences. These findings highlight the need for broader access to integration methods and tools to help users select the most appropriate one for their specific dataset.

Here, we present ‘SeuratIntegrate’, a flexible and comprehensive R package designed as an extension of Seurat by enabling seamless access to additional integration methods not natively supported in Seurat. In particular, it allows users to run both R- and Python-based methods entirely within the R environment. To date, SeuratIntegrate includes three R-based methods and five Python-based integration methods as well as complementary functions for performance evaluation.

## 2 Package description and implementation

### 2.1 Integration methods provided by SeuratIntegrate

SeuratIntegrate extends the functionality of Seurat v5 by providing access to additional integration methods not included in the original package, particularly those written in Python. Cross-language interoperability is handled internally by SeuratIntegrate, allowing users to access Python-based methods without leaving the R environment. The package performs in-RAM object conversion at runtime: relevant components of the Seurat objects are transformed into anndata format for compatibility with Python tools, and the resulting integration outputs (e.g. Assay, DimReduc, Neighbor or Graph) are automatically converted back and reintegrated into the Seurat object for downstream analysis. The “Post-processing” section of the vignette “Get started” provides additional guidance on this process. For integration methods that operate directly on gene count matrices and in line with findings in Luecken *et al.* (2022), SeuratIntegrate selects the most appropriate layer by default. Estimates of running time and memory usage for all supported integration approaches are available in the Supplementary Material, available as supplementary data at *Bioinformatics* online.

#### 2.1.1 Description of supported integration methods

SeuratIntegrate offers both R- and Python-based methods, enabling users to harness a wide range of computational approaches for dataset harmonization (Table 1). The expected input and output(s) of each integration method, including the most suitable data layer (e.g., raw counts, normalized expression), and the feature set to use (all genes or variable features), are listed in Supplementary Table S1, available as supplementary data at *Bioinformatics* online.

**Table 1.** Comprehensive overview of the integration methods provided by SeuratIntegrate.

Method	Type	Timing	Underlying algorithm	Reference
MNN	R	batchelor (Bioconductor)	Aligns datasets by mutual neighbors	Haghverdi *et al.* (2018)
Harmony	R	harmony (CRAN)	Iterative embedding correction	Korsunsky *et al.* (2019)
ComBat	R	sva (Bioconductor)	Empirical Bayes adjustment	Johnson *et al.* (2007)
scVI	Python	scvi-tools (GitHub, scverse/scvi-tools)	Variational autoencoder	Lopez *et al.* (2018)
scANVI	Python	scvi-tools (GitHub, scverse/scvi-tools)	Semi-supervised variational autoencoder	Xu *et al.* (2021)
BBKNN	Python	bbknn (GitHub, Teichlab/bbknn)	Batch-balanced nearest neighbors	Polański et al. (2020)
Scanorama	Python	scanorama (GitHub, brianhie/scanorama)	Manifold alignment	Hie *et al.* (2019)
trVAE	Python	scArches (GitHub, theislab/scarches)	Conditional variational autoencoder	Lotfollahi *et al.* (2020)

For each method, the following details are provided: original implementation language (R- or Python-based), link to the source code of this initial implementation, a brief description of the basic algorithm and reference to the original paper where the method was introduced. Acronyms: MNN—Mutual Nearest Neighbors; ComBat—Combating Batch Effects; scVI—single-cell Variational Inference; scANVI—single-cell ANnotation using Variational Inference; BBKNN—Batch balanced K Nearest Neighbors; Scanorama—Panoramic stitching of single-cell data; trVAE—transfer Variational AutoEncoder.

#### 2.1.2 Key functionality: DoIntegrate

Building on Seurat’s IntegrateLayers function introduced in version 5, SeuratIntegrate implements a new function called DoIntegrate. It provides users with:

- The ability to launch multiple integrations in a single command,
- Customizable parameters for each integration method,
- Flexibility in choosing the input matrix type and the features to include.

As highlighted by Luecken *et al.* (2022), the choice of input matrix can have a significant impact on integration performance. By allowing users to select the most appropriate matrix (raw, normalized or scaled) and features, DoIntegrate ensures flexibility in the use of integration workflows. Additionally, the function is compatible with other Seurat-supported integration methods, such as Canonical Correlation Analysis (CCA), Reciprocal Principal Component Analysis (RPCA), and FastMNN (available in SeuratWrappers), enabling seamless interoperability across different integration approaches.

#### 2.1.3 Reimplementation of Harmony and scVI

Although Harmony and scVI are already supported by Seurat, they have been re-implemented in SeuratIntegrate to provide users with enhanced flexibility:

- Harmony: Implemented via the RunHarmony function, adhering to the original developers’ guidelines. A seed is set by default to ensure reproducible results.
- scVI: Allows users to fine-tune specific parameters, such as the number of layers in the neural network and their size, providing greater control over the integration process.

#### 2.1.4 Python integration via reticulate

SeuratIntegrate leverages the reticulate package (Ushey *et al.* 2017) to enable Python-based integration methods. To streamline this process, the package includes tools to create and manage Conda environments for each Python-based method. UpdateEnvCache function offers a unified interface for creating, storing, and removing conda environments directly from R. We provide further guidance for troubleshooting common errors and issues with conda and reticulate in the “Troubleshouting with conda” section of the vignette dedicated to conda environments. Since R sessions can only load one Python environment at a time, SeuratIntegrate overcomes this limitation by using the Future package (Bengtsson 2021) to launch background R sessions for each environment. This functionality is seamlessly integrated into the DoIntegrate function.

For more details on using these features and additional functionalities, such as connectivity computation from cell-to-cell distances, please refer to the GitHub vignettes.

### 2.2 Evaluation metrics

#### 2.2.1 Metrics

**Figure 1.**
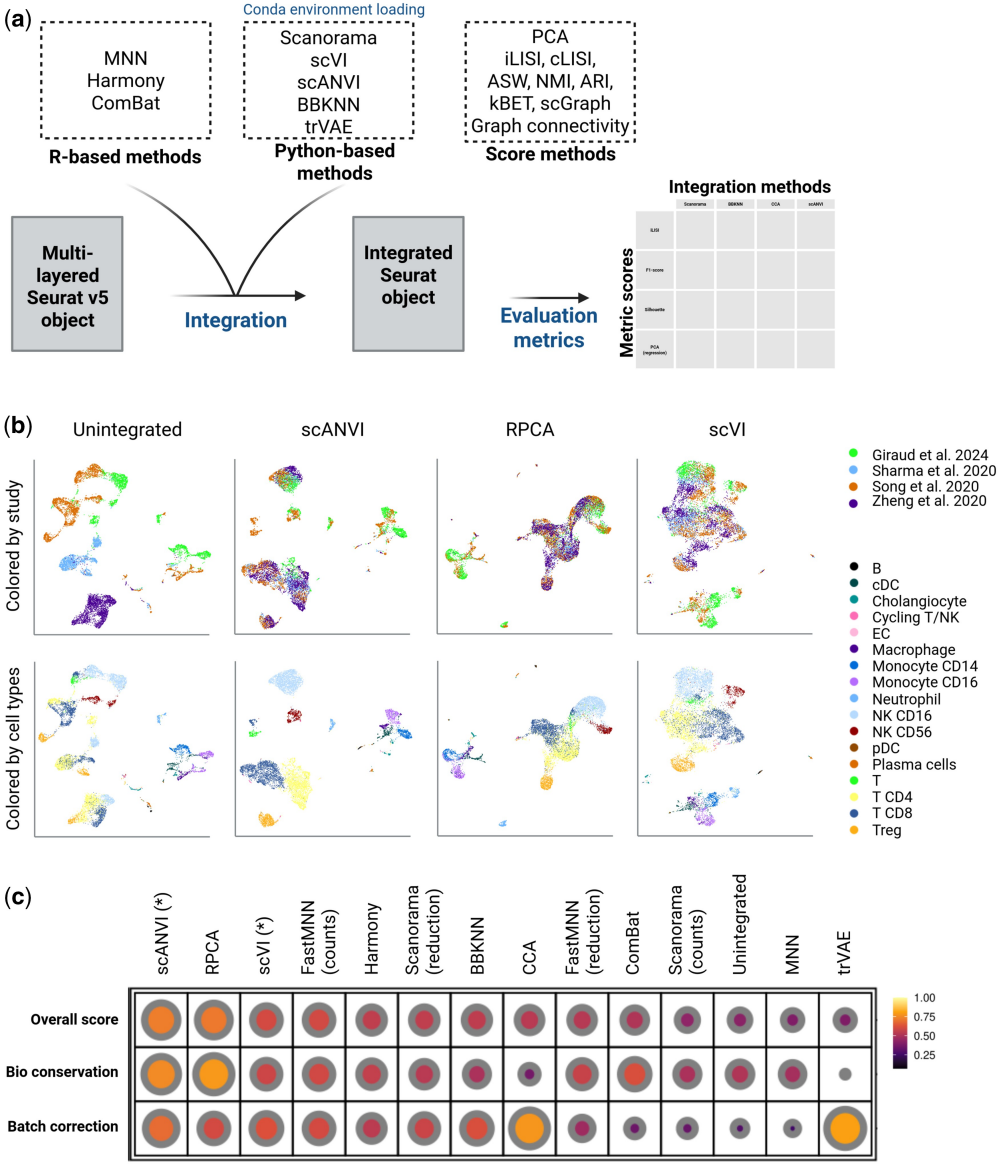
Overview of the SeuratIntegrate’s workflow. (a) Summary of methods available in SeuratIntegrate either for integration or evaluation of the integration. (b) Running example of the use of SeuratIntegrate with eight samples downloaded from four different studies, containing immune cells from liver tumor microenvironment. UMAPs from unintegrated, as well as best-performing scANVI, RPCA and scVI integrated data are colored by the origin of the study (upper) or by manual cell-type annotation (lower). (c) Dotplot displaying overall evaluation scores for all integration methods available in SeuratIntegrate. Absence of a dot indicates metrics that were not evaluated due to the characteristics of the integration method. For example, PCA scores are not computed for BBKNN because it returns a graph instead of numerical components. Label-based metrics were calculated using manually curated annotation with 17 cell types. Scores are organized in two main categories: batch correction and biological conservation. Asterisks (*) indicate methods guided with the cell-type labels during the integration.

SeuratIntegrate provides a suite of evaluation metrics to compare integration results (Fig. 1a), many of which have been previously described in Luecken *et al.* (2022) and Tran *et al.* (2020). These metrics include local inverse Simpson’s index (LISI) (Korsunsky *et al.* 2019) comprising iLISI and cLISI (Luecken *et al.* 2022), k-nearest neighbor batch effect test (kBET, Büttner *et al.* 2019), Regression-PCA (Büttner *et al.* 2019), cell-cycle conservation (Luecken *et al.* 2022), average silhouette width (ASW, Büttner *et al.* 2019, Luecken *et al.* 2022), graph connectivity (Luecken *et al.* 2022), normalized mutual information (NMI, Strehl and Ghosh 2002), adjusted Rand index (ARI, Hubert and Arabie 1985). We also included the recently published scGraph (Wang *et al.* 2024). Below, we group these metrics based on whether they require known cell-type labels or can be used in a label-free manner:

##### 2.2.1.1 Label-based metrics

- cLISI: Measures the preservation of cell-type purity.
- NMI and ARI: Focus on classification accuracy.
- Silhouette (ASW): Evaluates within- and between-cluster similarity and can be calculated with or without labels (batch-based).
- Graph Connectivity: Estimates to what extent same-label cells are directly connected in the k-nearest neighbor (KNN) graph.
- scGraph: Assesses preservation of between-cell-type distances within batches based on centroid distances.

##### 2.2.1.2 Label-free metrics

- iLISI and kBET: Measure batch mixing using graph-based methods. SeuratIntegrate allows users to adjust the parameter k for these metrics.
- PCA-related Scores:
- Regression-PCA: Quantifies batch removal via principal component regression.
- Density-PCA (DPCA): Uses Gaussian kernel density overlap as a proxy for batch removal.
- Cell-cycle conservation: Evaluates cell-cycle conservation before and after batch correction.

One can refer to the memo vignette covering scoring metrics to get a brief overview of input types expected by each score and the category it belongs to (either batch correction or bio-conservation).

#### 2.2.2 Scoring framework for integration evaluation and method selection

To streamline evaluation, SeuratIntegrate allows users to save multiple scores for different integration methods directly within the Seurat object as a two-dimensional array using functions starting with AddScore. All scores can be scaled between 0 and 1 for easier interpretation. Additionally, a batch-correction score and a bio-conservation score can be computed as a mean of the corresponding metrics. To ensure a more balanced contribution of each score, min–max rescaling can be performed beforehand, either on scores directly or on ranks computed from the scores. Finally, an overall score, calculated as the weighted sum of batch correction and bio-conservation scores for each method, is provided to guide users in selecting the best-performing integration.

The choice of integration method needs to consider several factors. Before integration, one can assess its necessity using simple visualization tools (e.g. PCA or Uniform Manifold Approximation and Projection (UMAP)), metrics like SCIntRuler (Lyu *et al.* 2024) or the PCA-regression score. While not quantitative, visual inspection of dimensional reductions is often a first step to detect major batch effects. Benchmarking studies, such as Luecken *et al.* (2022) can help guide initial method prioritization. Scalability tests (see Supplementary Material, available as supplementary data at *Bioinformatics* online) also provide valuable insights, especially for large datasets, where time and resource constraints may limit the range of feasible methods. When evaluating integration quality with SeuratIntegrate, rescaling ranks rather than raw scores tends to yield a more stable ranking of integrations (less prone to fluctuations when adding or removing integrations), with the top-ranked method typically preferred.

The final choice should reflect the user’s priorities—emphasizing batch correction, biological conservation, or both. After computing and rescaling scores, the top-ranked method based on these priorities can be selected. Its output can then be used for downstream analyses such as clustering, visualization, or differential expression within standard Seurat workflows.

#### 2.2.3 Visualization options

SeuratIntegrate includes three plotting options–dotplot, lollipop, and radar plots–within a unique function PlotScores to facilitate visual comparisons of integration performance. These plots are built using ggplot2, ensuring compatibility with popular visualization workflows.

## 3 Usage example

### 3.1 Dataset preparation

To demonstrate the utility of SeuratIntegrate, we analyzed immune cells from the hepatocellular carcinoma microenvironment. This dataset comprises eight samples from four publicly available studies: Sharma *et al.* (2020) [GSE156337], Song *et al.* (2020) [CRA002308], Zheng *et al.* (2020) [CRA001276], and Giraud *et al.* (2024) [GSE245909]. After preprocessing all samples (see Supplementary Material, available as supplementary data at *Bioinformatics* online), we randomly selected a total of 10 000 cells (2500 per dataset) and ran all eight integration methods available in SeuratIntegrate, as well as those implemented in Seurat.

### 3.2 Integration and evaluation

The unintegrated data revealed a clear bias in cell distribution based on the study of origin, as visualized in UMAP plots (Fig. 1b “Unintegrated” UMAP). For instance, CD4 T, CD8 T, and other T cells were segregated by study rather than biological cell type, highlighting the impact of batch effects and justifying the need for integration. Indeed, in a correct integration, we would expect cells to intermingle at the study level while remaining distinct by cell type.

Using SeuratIntegrate, we applied all integration methods and used the evaluation metrics including batch correction scores and biological knowledge conservation scores. Biological knowledge was assessed using a manual annotation containing 17 cell types (Fig. 1b—lower panels).

### 3.3 Findings

After integration, UMAP plots showed improved mixing of cells from different studies while preserving cell-type separation (Fig. 1b; Supplementary Material, available as supplementary data at *Bioinformatics* online), demonstrating the effectiveness of these methods in reducing batch effects and aligning datasets. The dotplot depicting evaluation metric scores revealed distinct performance patterns across different methods (Fig. 1c):

- Batch Correction: trVAE outperformed all methods, followed by CCA and scANVI. In contrast, MNN and unintegrated performed the worst.
- Biological Conservation: scANVI and RPCA ranked highest, followed by ComBat. trVAE and CCA integration yielded the lowest performance in this category.

Based on overall scores, scANVI provided the best integration performance, followed by RPCA and scVI.

## 4 Conclusion

The proposed R package named SeuratIntegrate facilitates the use of integration methods for single-cell transcriptomic data allowing easy interoperability between R and Python. We illustrated how SeuratIntegrate facilitates comprehensive integration and evaluation workflows by applying it to a dataset composed by immune cells from the hepatocellular carcinoma microenvironment. The observed variability in performance underscored the importance of dataset-specific evaluations, as demonstrated by our results and previous studies (e.g. Luecken *et al.* 2022).

## Supplementary Material

btaf358_Supplementary_Data

## Data Availability

The data underlying this article are available in the Zenodo Repository at https://dx.doi.org/10.5281/zenodo.14288360.
