# Supplementary material for: SeuratIntegrate: an R package to facilitate the use of integration methods with Seurat
Source: Bioinformatics. 2025 Jun 23;41(6):btaf358. doi: 10.1093/bioinformatics/btaf358 (PMC12206523; doi:10.1093/bioinformatics/btaf358)
Supplement: btaf358_Supplementary_Data [file btaf358_supplementary_data.pdf]

Supplementary material

SeuratIntegrate: an R package to facilitate the use of integration methods with Seurat

The supplemental material is structured in sections corresponding to those of the main manuscript.

Table of content

Integration methods and metrics....1
Scalability analysis of integration methods supported by SeuratIntegrate....2
    Conditions of scalability analysis execution....3
    Time, CPU and memory measurements....6
    Scalability results: Memory usage....6
    Scalability results: Runtime....7
Considerations for the choice of integration method....8
Supplemental information related to the Hepatocellular Carcinoma example....9
    Dataset preparation....9
    Integration results....10
Supplemental bibliography....12

Integration methods and metrics

This section aims to list the different integration methods and evaluation metrics available in SeuratIntegrate, with associated information such as required input types, preferred features or output types.

Supplementary Table 1. Integration methods either supported by or implemented in SeuratIntegrate. The expected input and output(s) are specified. When applicable, the type of count matrix (normalized or raw) and the type of features (all or variable) to use to achieve better performance is also specified. We added information about FastMNN, CCA and RPCA that are already available in Seurat v5. Further details regarding impacts of preferred feature selection can be found in Zappia *et al.* (2025).

**Integration method**	**Input type**	**Preferred layer**	**Preferred features**	**Output type**
ComBat	Counts	"data" (normalized)	Variable	Assay with corrected counts
Harmony	PCA	N/A	N/A	Dimensional reduction
MNN	Counts	"data" (normalized)	Variable	Assay with corrected counts
FastMNN	Counts	"data" (normalized)	Variable	• Assay with corrected counts<br>• Dimensional reduction
CCA	Counts	"data" (normalized)	Variable	Dimensional reduction
RPCA	Counts	"data" (normalized)	Variable	Dimensional reduction
BBKNN	KNN graph	N/A	N/A	KNN graph
Scanorama	Counts	"data" (normalized) or "counts" (raw)	All or variable	• Assay with corrected counts<br>• Dimensional reduction
scVI	Counts	"counts" (raw)	All	Dimensional reduction
scANVI	Counts	"counts" (raw)	All	Dimensional reduction
trVAE	Counts	"counts" (raw) or "data" (normalized) when recon loss is "mse"	All	Dimensional reduction

Supplementary Table 2. Scoring metrics for performance evaluation available in SeuratIntegrate. Metrics are listed with the input variables and the type of objects they require. The type of score to which they belong is indicated in the column 'Type'. For graph connectivity, a cell-label variable is only required when the `per.component` argument is set to `TRUE`.

Score		Cell type variable required	Clustering variable required	Input type
**Name**	**Type**			
Cell cycle regression	Bio-conservation			Dimensional reduction
PCA regression	Batch conservation			Dimensional reduction
PCA density	Batch conservation			Dimensional reduction
ASW batch	Batch conservation	✔		Dimensional reduction
ASW	Bio-conservation	✔		Dimensional reduction
scGraph	Bio-conservation	✔		Dimensional reduction
ARI	Bio-conservation	✔	✔	
NMI	Bio-conservation	✔	✔	
cLISI	Bio-conservation	✔		Dimensional reduction or KNN graph
iLISI	Batch conservation	✔		Dimensional reduction or KNN graph
kBET	Batch conservation	✔		Dimensional reduction or KNN graph
Graph connectivity	Batch conservation	✔ (`per.component = TRUE`)		KNN graph

Scalability analysis of integration methods supported by SeuratIntegrate

In this section, we evaluated the computational efficiency of the different integration methods by conducting scalability tests across datasets of varying sizes. We specifically measured execution time and memory consumption. These performance tests provide insights into methods' performance with increasing data size, demonstrating limitations and strengths of each method.

Dataset used to perform scalability analysis

The scalability test was performed on the "Human Immune Health Atlas" by the Allen Institute (Gong A., et al, 2024). This dataset contains 1,821,725 cells divided into 36 sequencing batches counting 10,217 to 146,394 cells each. The `immune_health_atlas_full.h5ad` file was downloaded and loaded using Scanpy. Each batch's raw counts were converted to mtx files, which were then used to create `SeuratObjects` for each scaling analysis run.

Supplementary Table 3. List of batches used in the variable sized datasets. Each dataset (10, 50, 100, 250, 500 and 1,000 thousand cells) were built by randomly sampling cells in the smallest batches.

**Number of cells**	**Randomly sampled batches**
10,000	B055, B132
50,000	B132, B067, B082, B085
100,000	B055, B132, B067, B082, B085, B142, B007, B010
250,000	B055, B132, B067, B082, B085, B142, B007, B010, B043, B064, B050, B145, B036, B080
500,000	B055, B132, B067, B082, B085, B142, B007, B010, B043, B064, B050, B145, B036, B080, B084, B047, B063, B054, B060, B039
1,000,000	B055, B132, B067, B082, B085, B142, B007, B010, B043, B064, B050, B145, B036, B080, B084, B047, B063, B054, B060, B039, B048, B096, B138, B045, B001, B077, B073, B056, B094

Conditions of scalability analysis execution

We performed two rounds of scalability tests with different number of threads:

- 4 threads
- 10 threads

The implementation of integration methods included in the test differ in their parallelization approaches. To ensure that the allowed number of simultaneous threads was not exceeded, we adapted monitoring when necessary. Further details are given in Supplementary Table 4.

Moreover, few integration methods could not be run, because of time or available memory limitations. Those are specified in Supplementary Table 5.

Supplementary Table 4. Parallelization control means used for each integration method. Green check marks designate parallelization strategies that we are using, while red crosses correspond to enforced single-threading. With the exception of the first one, the column names consist of the parallelization strategy and the monitoring package (in brackets).

Integration method	Forking with MulticoreParam (`BiocParallel`)	Forking with multicore backend (`future`)	BLAS & OMP (`RhpcBLASctl`)	OMP or MKL intra-op parallelism (`Torch`)
**ComBat**	✔			
**Harmony**			✔	
**FastMNN**	✔			
**RPCA**		✔	✘	
**CCA**		✔	✘	
**BBKNN**			✔	
**Scanorama**			✔	
**scVI**				✔
**scANVI**				✔
**trVAE**				✔

Supplementary Table 5. Summary of integration methods included in the scalability tests, according to the number of cells in the datasets and the allowed number of threads. The green check mark and the red cross respectively indicate inclusion or exclusion of the integration method in the scalability analysis, depending on the size of the dataset.
MNN was excluded from the scalability assessment due to its prohibitively long runtime.
For the 1 million cells subsampling, CCA integration's memory usage exceeded the system's RAM limitation on 4 threads. With 10 threads, RPCA failed for the same reason. We therefore limited the scalability test to 4 threads for 1 million cells.
In the "Number of threads" column, specifically, a green check mark emphasizes a successful run.

**Number of cells**	**Number of threads**		**ComBat**	**Harmony**	**MNN**	**FastMNN**	**RPCA**	**CCA**	**BBKNN**	**Scanorama**	**scVI**	**scANVI**	**trVAE**
	4	10											
10,000	✔	✔	✔	✔	✘	✔	✔	✔	✔	✔	✔	✔	✔
50,000	✔	✔	✔	✔	✘	✔	✔	✔	✔	✔	✔	✔	✔
100,000	✔	✔	✔	✔	✘	✔	✔	✔	✔	✔	✔	✔	✔
250,000	✔	✔	✔	✔	✘	✔	✔	✔	✔	✔	✔	✔	✔
500,000	✔	✔	✔	✔	✘	✔	✔	✔	✔	✔	✔	✔	✔
1,000,000	✔	N/A	✔	✔	✘	✔	✔	✘	✔	✔	✔	✔	✔

Time, CPU and memory measurements

All scalability tests were launched on a HPC cluster node with two CPU sockets counting 16 single-threaded physical cores each (32 threads in total) and 1007.68 GiB of RAM. Global memory usage and process-wise statistics were probed every 5 seconds. Memory usage was reported by the `free` command-line tool and processes were recorded by the `top` command. Both are from the procps-ng package version 3.3.10. All tests were run from the terminal with nohup (GNU coreutils 8.22).

Scalability results: Memory usage

This section summarizes results of memory usage throughout the scalability test, across the different integration methods.

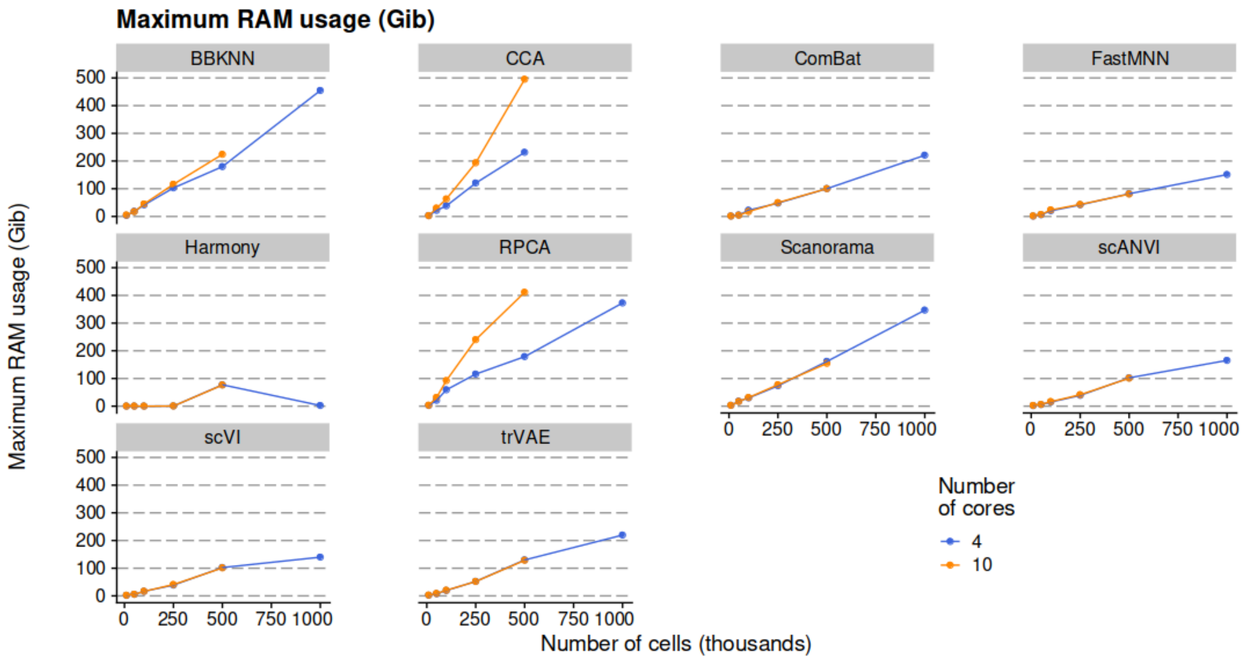

Supplementary Figure 1. Maximum amount of memory (in Gib) reported for each integration method, according to the number of threads available and the number of cells in the dataset. Each panel aggregates the results of a specific integration method, but they all share the same x- and y-axes scales. Memory usage is on the y-axis and the x-axis is scaled by the number of cells in the dataset. Lines and dots are colored according to the number of threads, blue and orange correspond to 4 and 10 cores respectively.

Supplementary Table 6. Summary of the maximum memory usage peak probed during the entire scalability test runs, depending on the size of the dataset, the numbers of batches and threads.

Number of cells	Number of batches	Maximum peak of occupied RAM (GiB)	
		4 threads	10 threads
10,000	2	9.33623886108398	9.81008529663086
50,000	4	39.488639831543	47.4854469299316
100,000	8	90.1255187988281	118.127464294434
250,000	14	204.758422851563	299.425659179687
500,000	20	397.598850250244	658.688652038574
1,000,000	29	774.952266693115	N/A

Scalability results: *Runtime*

This section summarizes results of running time throughout the scalability test, across the different integration methods.

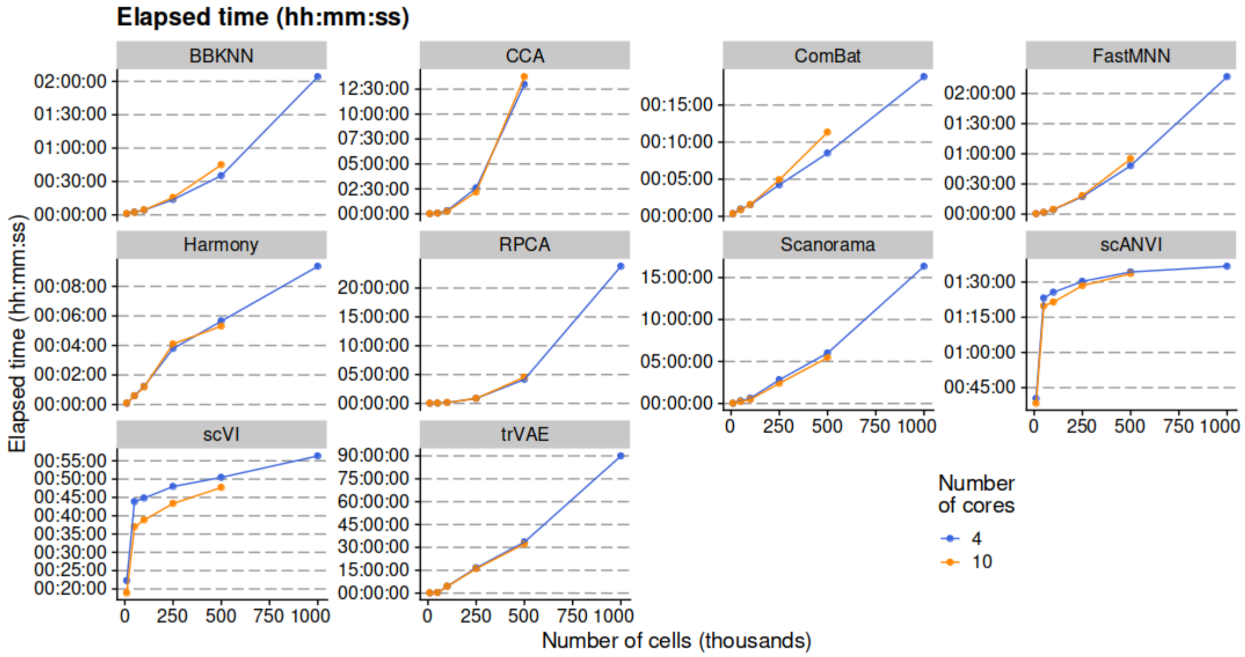

Supplementary Figure 2. Elapsed time (in hours:minutes:seconds) for each integration method, according to the number of threads available and the number of cells in the dataset. Each panel aggregates the results of a specific integration method. Y-axes indicate elapsed time, specific to each integration method. X-axes, common to all integration methods, represent the number of cells in the datasets. Lines and dots are colored according to the number of threads, blue and orange correspond to 4 and 10 cores respectively.

Supplementary Table 7. Summary of the total amount of time elapsed throughout scalability test runs, depending on the size of the dataset, number of batches and number of used cores.

Number of cells	Number of batches	Total time (hh:mm:ss)	
		4 threads	10 threads
10,000	2	01:18:28.446393	01:11:46.106392
50,000	4	03:11:11.669904	02:51:12.895052
100,000	8	08:13:28.128701	07:44:22.697721
250,000	14	26:22:54.270791	24:56:33.777502
500,000	20	61:50:08.516482	61:38:48.197833
1,000,000	29	140:01:31.177622	N/A

Considerations for the choice of integration method

Selecting the optimal integration method from numerous available ones is a significant challenge. In this regard, several parameters are to be taken into account to make an informed choice.

First and foremost, the pertinence of undertaking an integration should be evaluated. The SCIntRuler metric implemented in the eponymous R package or the PCA-regression score available with SeuratIntegrate can provide insights. A visual inspection of low dimensional reductions - PCAs, UMAPs or t-SNEs - is a complementary albeit more error-prone and subject to bias.

If an integrative analysis seems appropriate, publications presenting benchmarking results or SCIntRuler can provide a valuable starting point for method prioritization. As the number of cells increases, time and computing requirements can also limit the panel of methods worth considering. The scalability tests presented in the previous section is also a helpful resource.

For the final selection with the scoring metrics included in SeuratIntegrate, we recommend to rescale the ranks rather than the scores directly, as it is more stable (i.e. the final ranking of integrations fluctuates less when adding or removing integrations). In most cases, the integration with the highest overall score should be favoured over the others. But the score profile of integrations can also provide resourceful information. If a user is particularly interested in a specific subset of scores, they can either compute the overall scores on this restricted set or favor the integration that obtains the best scores in the set among the global top 10-20%. In the case of our example, RPCA better preserves cell cycle and reaches a higher kBET rank than scANVI despite ranking second on the overall score scale (Fig. 1c and Supplementary Fig. 4). Furthermore, a user particularly interested in batch-effect removal (or bio-conservation) can increase its weight in the overall score which is 40% (60%) by default. Nonetheless, it is not

recommended to completely ignore one of them. We also discourage using low dimensional reduction comparison in the selection process, especially UMAPs or t-SNEs, because they do not provide robust insight, and they can be tuned later, after choosing the most promising integration. In contrast, we warmly recommend selecting the integration with the highest overall score or at least one in the top 10%.

Supplemental information related to the Hepatocellular Carcinoma example

Dataset preparation

As a biological example, we investigated a dataset comprising eight samples from four publicly available studies: Sharma et al. (2020) [GSE156337], Song et al. (2020) [CRA002308], Zheng et al. (2020) [CRA001276], and Giraud et al. (2024) [GSE245909]. All samples were generated using 10x Genomics technology and sequenced on an Illumina platform. After downloading the raw data, we used CellRanger v7.1.0 to generate raw gene count matrices, followed by cell quality filtering, doublet and non-immune cell removal, and merging into a multi-layered Seurat object (~40,000 cells). For integration, we randomly selected 10,000 cells to speed up the analysis. Scripts to generate the example object as well as the multi-layered object are available on a Zenodo deposit (accession 10.5281/zenodo.14288360).

Integration results

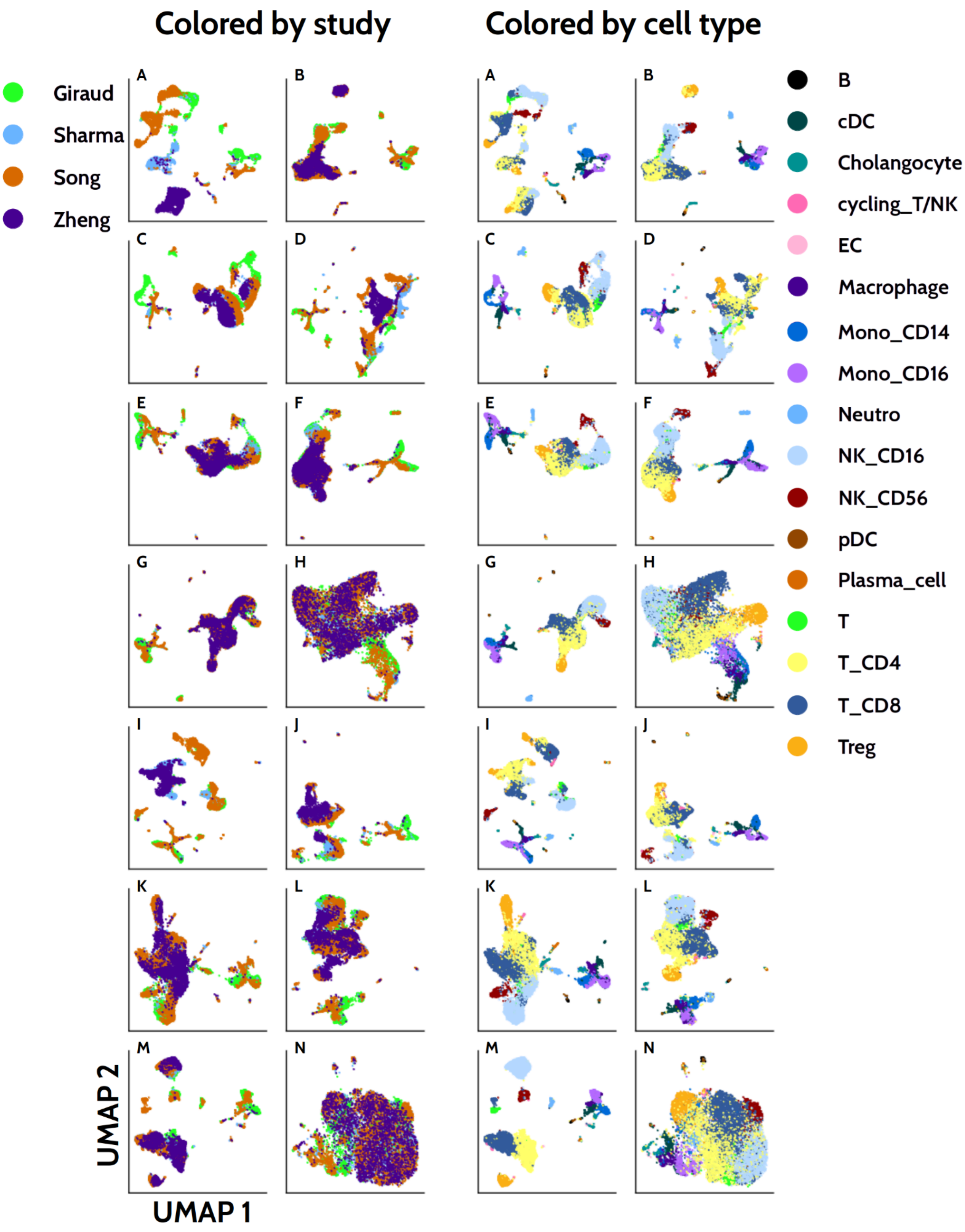

Supplementary Figure 3. UMAPs obtained without and with integration. Cells are colored by study of origin on the left panels, and by cell type on the right ones. Identical letters on the right and left panels identify the same UMAP, different letters correspond to distinct integration (or non-integrated) outputs. A) Unintegrated, B) Harmony, C) ComBat, D) MNN, E) FastMNN (reduction), F) FastMNN (counts), G) RPCA, H) CCA, I) Scanorama (counts), J) Scanorama (reduction), K) BBKNN, L) scVI (*), M) scANVI (*), N) trVAE. Asterisks (*) indicate methods guided with the cell-type labels during the integration.

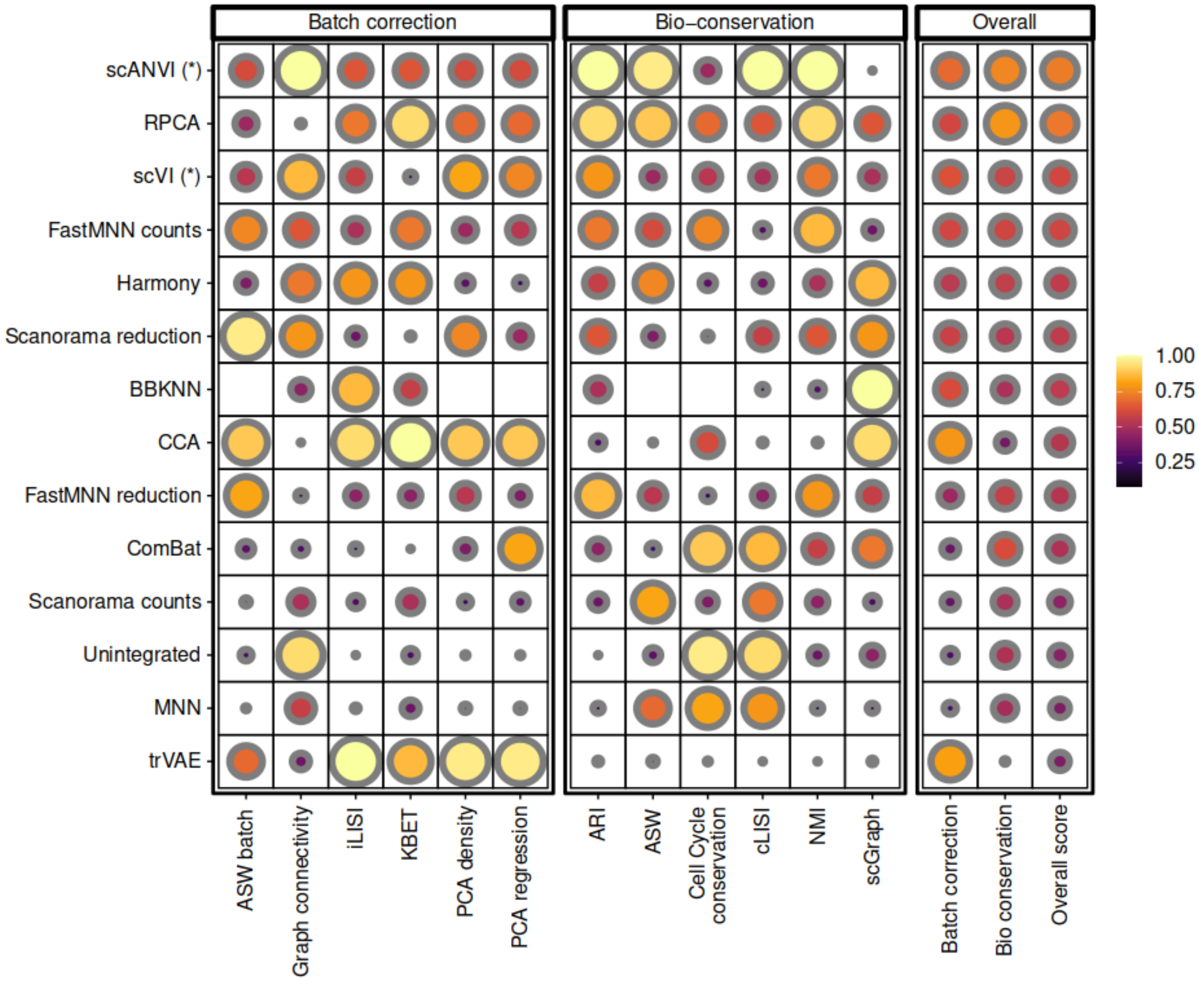

Supplementary Figure 4. Scores obtained with evaluation metrics available in SeuratIntegrate. Integration outputs are ordered on the y-axis by decreasing overall score. Evaluation metrics on the x-axis are organized by type ("Batch correction", "Bio-conservation" or "Overall"). Batch correction and Bio-conservation scores were obtained by applying min-max rescaling on ranks of scores scaled between 0 and 1. Overall batch correction and bio-conservation scores are the mean of scores from the corresponding type. Absence of a dot indicates metrics that were not evaluated due to the characteristics of the integration method. For example, PCA scores are not computed for BBKNN because it returns a graph instead of numerical components. Label-based metrics were calculated using manually curated

annotation with seventeen cell types. The size and color of each circle reflect the ranking of the integration output for a particular score. Larger circles with lighter color indicate better performance. Asterisks (*) indicate methods guided with the cell-type labels during the integration.

Supplemental bibliography

Gong Q, Sharma M, Kuan EL *et al*. Longitudinal Multi-omic Immune Profiling Reveals Age-Related Immune Cell Dynamics in Healthy Adults. bioRxiv. 2024. p. 2024.09.10.612119. DOI: 10.1101/2024.09.10.612119.

Zappia L, Richter S, Ramírez-Suástegui C *et al*. Feature selection methods affect the performance of scRNA-seq data integration and querying. *Nat Methods*. 2025 Apr;22(4):834-844. DOI: 10.1038/s41592-025-02624-3.
